# A RANDOMIZED CLINICAL TRIAL OF ONLINE SOCIAL INTELLIGENCE TRAINING FOR ADOLESCENT CUSTODIAL GRANDCHILDREN

**DOI:** 10.1093/geroni/igae098.2723

**Published:** 2024-12-31

**Authors:** Megan Dolbin-MacNab, Gregory Smith, Luxin Hu, Frank Infurna

**Affiliations:** Virginia Tech, Blacksburg, Virginia, United States; Kent State University, Kent, Ohio, United States; Kent State University, Kent, Ohio, United States; Arizona State University, Tempe, Arizona, United States

## Abstract

Although custodial grandchildren (CG) experience multiple negative psychosocial outcomes, intervention research on this population has been virtually absent. To address this gap, we conducted a two-arm randomized clinical trial (RCT), which compared the efficacy of online Social Intelligence Training (SIT) to an attention control condition, at enhancing psychological and relational outcomes among a sample of 349 adolescent (ages 11-18 years) CG. Online surveys were self-reported at baseline (T1), post-intervention (T2), and at 3- (T3), 6- (T4), and 9- (T5) months. Latent change score analyses revealed significant differences in favor of SIT for CG internalizing difficulties (T4) and self-esteem (T2; T4). Relationally, significant differences in favor of SIT were found for CG prosocial behavior (T5) and avoidant (T2) and anxious (T2) attachment to the grandmother. Across outcomes, effect sizes ranged from.28 to.47. Although the treatment effects were modest, subsequent CART analyses identified that treatment-related change in the SIT condition was predicted by the baseline values of each respective latent outcome (effect sizes =.51 – 1.53). In turn, ANOVAs revealed that changes in latent outcomes from T1 to T2 were significantly moderated by their respective baseline values (p =.05 –.002) for externalizing and internalizing difficulties, self-esteem, and avoidant attachment to the grandmother. The only significant moderator at T5 was the baseline value of support from friends (p =.03). We conclude that RCTs which only report average treatment effects, without also investigating potential moderators of differential treatment efficacy, may underestimate true intervention impact. [Funded by R01AG054571)].

